# Beyond the Brackets: A Case Report of Acute Angioedema Associated With Orthodontic Treatment

**DOI:** 10.7759/cureus.95018

**Published:** 2025-10-20

**Authors:** Akansha Pandey, Ajit V Parihar, T P Chaturvedi, Dishant Mahajan, Vedita Singh

**Affiliations:** 1 Department of Orthodontics, Faculty of Dental Sciences, Institute of medical sciences, Banaras Hindu University, Varanasi, IND; 2 Department of Orthodontics, Faculty of Dental Sciences, Institute of Medical Sciences, Banaras Hindu University, Varanasi, IND; 3 Department of Orthodontics, Faculty of Dental Sciences, Institute of Medical Sciences, Banaras Hindu University, varanasi, IND; 4 Department of Oral and Maxillofacial Surgery, Faculty of Dental Sciences, Institute of Medical Sciences, Banaras Hindu University, Varanasi, IND

**Keywords:** allergic reaction, angioedema, dental material, etchant, orthodontic treatment

## Abstract

This case report highlights a rare incidence of acute angioedema triggered by an etchant during orthodontic treatment. It underscores the potential for commonly used dental materials to provoke allergic reactions. A 14-year-old female patient developed lip swelling, burning sensation, and localized pain shortly after etching. The reaction involved predominantly the upper lip, and the patient had no known history of allergy. Clinical signs included diffuse, non-pitting edematous swelling of the lips without systemic involvement. Acute allergic angioedema was diagnosed based on clinical evaluation. Immediate treatment with IV antihistamines and corticosteroids led to complete resolution. Chemical agents used in orthodontics can trigger hypersensitivity reactions. Early recognition, prompt management, and preventive strategies are essential to ensure patient safety during dental procedures.

## Introduction

Acute angioedema is characterized by a sudden onset of localized, transient swelling of the subcutaneous or submucosal tissues, most frequently involving the face, lips, tongue, pharynx, and extremities [1]. Its severity can range from mild, self-limiting episodes to life-threatening manifestations that may compromise the airway and require urgent intervention [2]. Although acute angioedema associated with orthodontic treatment is uncommon - reported in approximately 0.3-0.4% of individuals undergoing dental care, with an estimated frequency of 1 in 700 to 1 in 2,600 dental procedures - hypersensitivity reactions to dental materials, including bonding agents and etchants, are increasingly recognized [3]. Dental etchants commonly contain 37% phosphoric acid as the active ingredient along with excipients such as carboxymethyl cellulose (CMC) to improve viscosity and application properties [4]. The use of 37% phosphoric acid in conventional acid-etching systems remains the standard technique for bonding metallic orthodontic brackets [5]. While generally considered inert, excipients such as CMC can act as allergens or irritants by triggering immediate hypersensitivity reactions through IgE-mediated pathways. This highlights the importance of considering not only the active ingredients but also the excipients when evaluating adverse events related to dental materials. This case report highlights a rare complication of acute angioedema during orthodontic therapy, underscoring the necessity of clinical attentiveness to early allergic reactions, careful evaluation of hypoallergenic components, and prompt management to ensure patient safety.

## Case presentation

The study was conducted in accordance with CARE guidelines [6], and written informed consent was obtained from the patient for treatment and for publication of the case and accompanying images. All patient data were de-identified. Ethics approval was not deemed necessary due to the case report’s study design.

Case history

A 14-year-old female patient presented with a chief complaint of irregular tooth alignment. The patient had no prior medical, dental, or allergy history. Clinical examination and diagnostic records indicated an Angle’s class I malocclusion with increased overjet and crowding in the lower arch. A comprehensive orthodontic treatment plan involving extractions was developed; however, no extractions had been carried out before the bonding procedure.

Procedure

A comprehensive fixed orthodontic treatment plan was developed to improve her soft tissue profile and correct the dental malocclusion. The initial phase of treatment involved etching the tooth surfaces with 37% phosphoric acid to prepare for bracket bonding. During the etching process, the patient’s upper lip mucosa came into contact with the etchant applicator tip, leading to unintended exposure. Immediate action was taken by thoroughly rinsing the affected area with water to neutralize the acid and prevent tissue damage, and the etchant contact incident was addressed promptly. The procedure was then resumed, and brackets were successfully bonded using light-cure adhesive.

Immediate symptoms 

**Figure 1:**
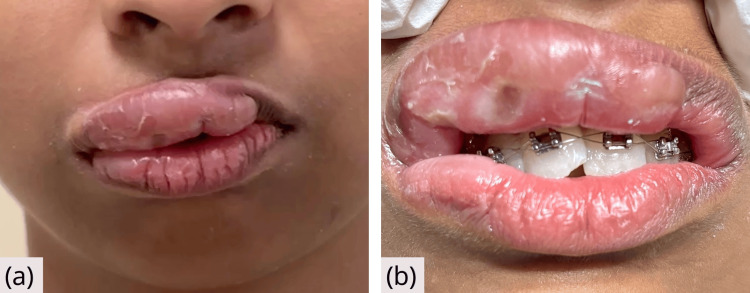
Clinical presentation of upper lip angioedema 30 minutes after exposure to the allergen (a) Extraoral photograph showing swelling of the upper lip consistent with acute angioedema. The swelling is predominantly localized to the right upper lip and the right commissure of the lower lip, with slight extension across the midline of the upper lip. (b) Intraoral view revealing edematous upper labial mucosa 30 minutes after exposure.

Approximately 30 minutes post-procedure, the patient reported a burning sensation on the lips, followed by localized swelling involving the right upper lip and the right commissure of the lower lip, with slight extension across the midline of the upper lip (Figure 1). The patient was closely observed for any progression of symptoms.

Diagnostic assessment

The diagnosis involved a comprehensive approach that combined clinical evaluation, along with blood investigation and allergy tests. Careful documentation of symptoms was made, including their onset, duration, and progression. The diagnostic algorithm for suspected drug hypersensitivity was followed [7].

The patient was conscious, and the airway was clear. The body temperature was 36.7°C, blood pressure was 128/72 mm Hg, pulse rate was 80 beats per minute, and SpO_2_ was 99%. No sign of dyspnea was seen. A swelling was seen on the upper lip. On palpation, the swelling was soft and edematous, with no sign of erythema, pain, or tenderness. No sign of erythema or swelling was noted on the gums or vestibule. The onset was sudden, and the swelling gradually increased in size. The blood examination revealed no abnormality other than a mildly raised eosinophil count (6%). A skin patch test was done to identify the potential allergic trigger.

The acute nature of the swelling and raised eosinophil count suggested a localized type I hypersensitivity reaction, specifically acute allergic angioedema. The blood investigation was although not very conclusive. To search for the allergen, a skin patch test was done by applying patches of the potential allergen on the forearm skin, such as etchant, bonding agent, and adhesive, which were used during the procedure. The result was noted after 24-48 hours [7].

Therapeutic intervention

The patient was promptly informed, and thorough counselling was provided to address concerns. Immediate airway assessment was performed to detect any signs of respiratory distress or vocal changes. Pharmacological intervention involved the administration of an IV antihistamine (pheniramine malate 2 mL [1 mL contains 22.75 mg]) and systemic corticosteroid (dexamethasone 8 mg). As part of supportive care, the patient was advised to apply cold compression pack to the affected area to alleviate discomfort and minimize swelling. Emphasis was placed on maintaining adequate hydration to ensure proper fluid balance. A follow-up was scheduled after 24 hours to evaluate progress.

Follow-up and outcome

**Figure 2:**
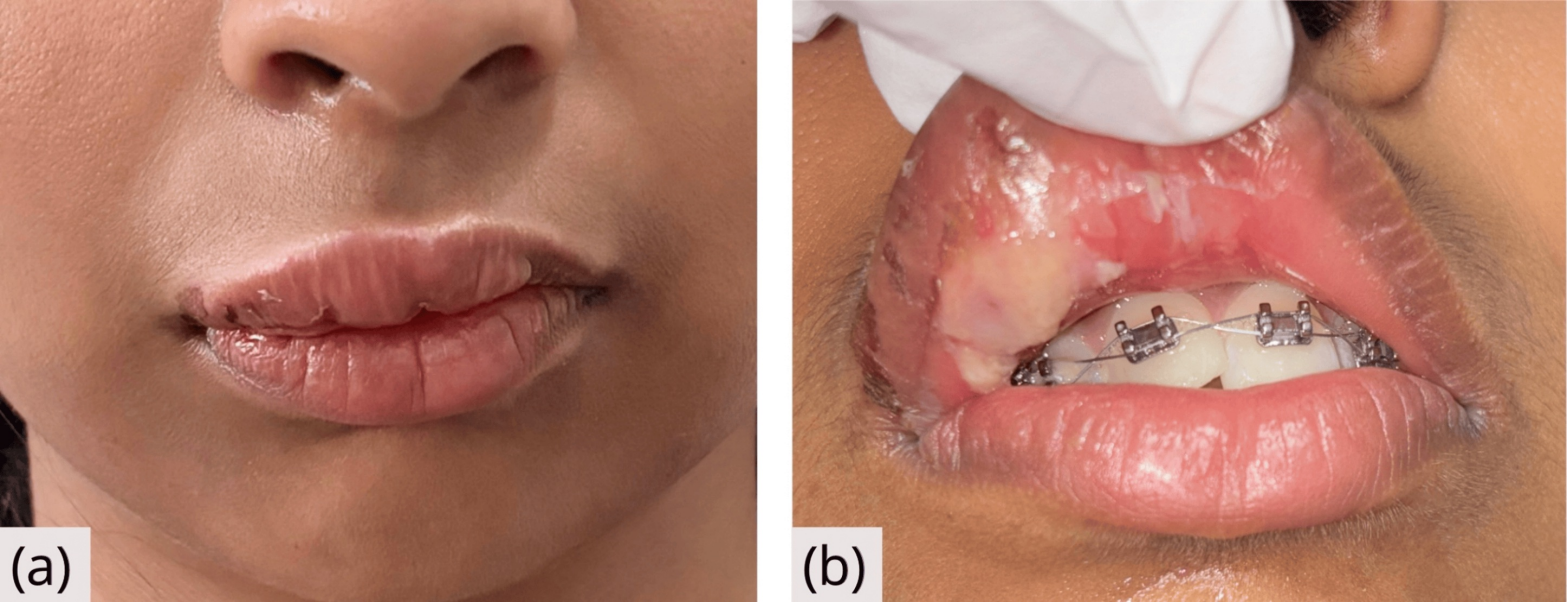
Follow-up after 24 hours (a) Extraoral photograph showing reduction in swelling of the upper lip after pharmaceutical treatment. (b) Intraoral view showing necrotic changes of the upper lip mucosa at the site of exposure.

After 24 hours, the lip swelling reduced significantly, and intraorally, localized mucosal necrosis was seen (Figure 2). No pain, tenderness, or erythema was present. Topical corticosteroid gel (triamcinolone acetonide 0.1%) was prescribed to be applied to the intraoral lesion.

**Figure 3:**
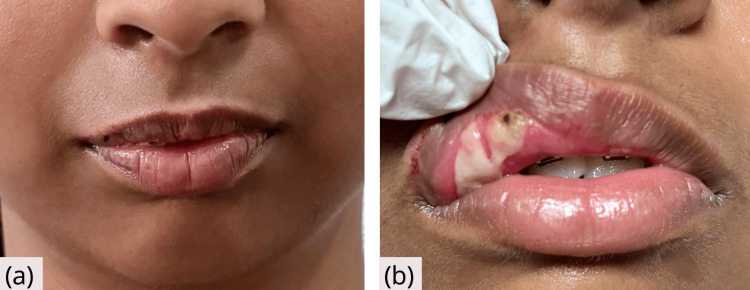
Follow-up after 48 hours (a) Extraoral photograph showing marked reduction in swelling and restoration of normal lip contour. (b) Intraoral view showing slough formation on the upper labial mucosa at the site of exposure.

After 48 hours, the lip swelling diminished, and intraorally, there was a well-demarcated region of slough formation (Figure 3). The patch applied to the forearm skin was removed, and the reaction was noted. Mild erythema was observed at the area exposed to the etchant, but as no wheal or elevated lesion was evident, the reaction could not be considered positive. No reaction was noted with the adhesive and bonding agent. The previously published study reported that when allergens are absorbed through the oral mucosa, symptoms are more rapid and more severe than when they are absorbed through the skin [8]. In the present case, the causative agent was presumed to be absorbed from the oral mucosa, causing a strong allergic reaction and lip swelling similar to acute symptoms.

**Figure 4:**
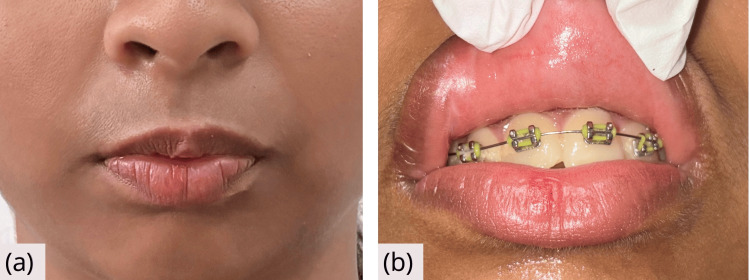
Follow-up after 10 days (a) Extraoral photograph showing complete resolution of swelling. (b) Intraoral view showing complete healing with no scar.

After 10 days, complete healing was noted. No scar mark was noted on the lips and intraoral mucosal tissue (Figure 4).

## Discussion

In 1955, Buonocore introduced enamel etching with 85% phosphoric acid [9], pioneering a method for bonding acrylic structures through micromechanical retention. This process involves dissolving hydroxyapatite crystals to create an irregular enamel surface that allows adhesive penetration [10]. Today, 37% orthophosphoric acid is commonly used for 30 seconds to achieve similar results [11]. Composition of dental etching gel constitutes phosphoric acid in aqueous solution form, a viscosity-enhancing agent, CMC, and optionally additives [12]. Even brief exposure to phosphoric acid can cause tissue necrosis and localized erythema. While CMC is generally considered a safe and common excipient to thicken and stabilize dental etchant [4], it can trigger allergic reactions in some individuals, as reported [13-15]. To reveal the hidden culprit in the etchant composition, provocation test should be done after careful assessment of risk-benefit ratio [16].

In this case, nickel allergy was ruled out because the mechanism underlying nickel hypersensitivity involves a type IV delayed hypersensitivity reaction, which is mediated by T-cells [17]. Nickel hypersensitivity may present not only as a localized reaction but also as a trigger for systemic manifestations [18,19].

The pathogenesis of type 1 hypersensitivity reaction involves an immune system response to an allergen, typically mediated by IgE antibodies. Upon exposure to the allergen, IgE binds to receptors on mast cells and basophils, triggering their degranulation. This releases histamine, prostaglandins, and leukotrienes, which increase vascular permeability and cause inflammation [20]. The resulting symptoms, such as swelling, redness, itching, and, in severe cases, airway obstruction, occur rapidly and may progress without timely intervention.

Allergic angioedema typically appears rapidly after exposure to a triggering factor, such as food intake, Hymenoptera sting, or medication use, and usually resolves quickly with the administration of antihistamines and epinephrine, without recurrence unless re-exposure to the allergen occurs [21].

Effective management of hypersensitivity reactions in orthodontic patients should involve a tri-focal approach: prevention, symptom management through pharmacological and non-pharmacological interventions, and patient education.

1. Prevention is the most important treatment for allergic responses, which includes contact avoidance and elimination of the offending agents.

2. Pharmacological treatments include topical corticosteroids to reduce inflammation locally and antihistamines to manage type I hypersensitivity symptoms such as itching and swelling [22]. Systemic corticosteroids may be used for severe cases under careful monitoring. Non-pharmacological measures focus on excellent oral hygiene, dietary modifications to avoid irritating foods, and promoting healing. Patients should follow gentle oral care routines and use non-irritating mouth rinses.

3. Patient education is critical, emphasizing awareness of triggers and allergens in dental materials, medications, and foods. For individuals with a known history of angioedema, the indication for prophylactic measures prior to a procedure is guided by the specific diagnosis [22].

Limitations and future direction

Prior to orthodontic treatment, thorough assessments including medical history and allergy testing are essential for identifying potential allergens. Although rarely conducted, histopathologic examination of oral lesions can help differentiate hypersensitivity reactions from similar pathologies. For patients with known angioedema, prophylaxis may be appropriate, and any identified allergens should be clearly documented. Future efforts should focus on developing more biocompatible orthodontic materials [23] and identifying genetic markers linked to hypersensitivity. Advancements in materials science and immunology are key to reducing allergic reactions and improving patient outcomes.

## Conclusions

This case underlines the importance of being alert to possible hypersensitivity reactions during orthodontic care. While the clinical presentation is suggestive of a type I hypersensitivity reaction, a definitive etiology cannot be established without further evaluation. CMC or the phosphoric acid in the etchant may be considered a potential trigger; however, their role remains speculative in the absence of confirmatory testing. To establish a causal relationship, additional investigations such as provocation testing or immunologic assessments would be required. Even a commonly used material such as etchant can, in rare situations, trigger acute allergic responses such as angioedema. Recognizing the signs early, managing them promptly, and taking preventive steps such as reviewing materials and assessing patient history are key to ensuring patient safety and effective treatment.
